# Whole-genome sequence of 19 *Listeria monocytogenes*, isolated from municipal wastewater in State College, Pennsylvania

**DOI:** 10.1128/mra.00523-25

**Published:** 2025-09-05

**Authors:** Erin Horack, Anna Acosta, Matthew Jones, Jie Feng, Edwin Omar Rivera-Lopez, Nkuchia M. M'ikanatha, Jasna Kovac, Edward Dudley

**Affiliations:** 1 Department of Food Science, The Pennsylvania State University 8082, University Park, Pennsylvania, USA; 2 Department of Biology, The Pennsylvania State University 8082, University Park, Pennsylvania, USA; 3 Division of Infectious Disease Epidemiology, Pennsylvania Department of Health 6616, Harrisburg, Pennsylvania, USA; University of Maryland School of Medicine, Baltimore, Maryland, USA

**Keywords:** *Listeria monocytogenes*, wastewater-based surveillance, genomics, food-borne pathogens, food safety

## Abstract

Eight wastewater samples were collected from three sites in State College, Pennsylvania, during June and July 2024. Nineteen *Listeria monocytogenes* were isolated and sequenced. Three isolates were ≤9 SNPs different from human cases deposited in GenBank, highlighting the potential for wastewater-based surveillance to monitor *L. monocytogenes* outbreaks.

## ANNOUNCEMENT

Wastewater-based surveillance (WBS) is a powerful tool for public health monitoring, offering a more comprehensive view of pathogen prevalence than traditional methods (1–6). *Listeria monocytogenes*, a major foodborne pathogen, infects ~1,600 Americans annually, leading to 260 deaths (7).

As part of a broader effort to monitor foodborne pathogens in wastewater, *L. monocytogenes* was isolated from three wastewater treatment facilities in State College, PA: Pennsylvania State University’s Water Reclamation Facility (Campus), University Area Joint Authority (UAJA); and manhole BD1100, which receives wastewater from downtown State College before reaching UAJA facility (Downtown). Microorganisms were collected by centrifugation and vacuum filtration (Fisher Scientific, 0.45 µm membrane) (4). Pellets and filters were incubated in 225 mL of buffered *Listeria* enrichment broth (VWR International, LLC) containing sodium pyruvate (1.1 g/L) at 30°C for 4 h, followed by supplementation with nalidixic acid (40 mg/L), cycloheximide (50 mg/L), and acriflavine hydrochloride (10 mg/L), and incubation at 30°C for 24–48 h. Enrichments were streaked onto *Listeria* Ottaviani and Agosti (Sigma-Aldrich) and Rapid L’mono (Bio-Rad) agars, incubated for 48 h, then subcultured on Brain Heart Infusion agar (VWR International, LLC) at 37°C for 24 h. Following incubation, isolated cultures were transferred to Luria-Bertani broth (VWR International, LLC) and incubated at 37°C for 24 h. DNA was extracted using the Qiagen DNeasy Blood and Tissue Kit (8). Presumptive isolates were confirmed using PCR targeting *iap* (*Listeria* spp.) and *lmo2234* (*L. monocytogenes*) (9).

Confirmed isolates were sequenced using an Illumina MiSeq (Illumina, Inc.) with MiSeq Reagent Kit v3, 600 Cycles, prepared using the Illumina DNA Prep Kit (Illumina, Inc.) (10).

Read quality and sequence type were confirmed using MicroRunQC workflow (v1.2) in GalaxyTrakr (11, 12). Quality control metrics included average read quality ≥30, coverage ≥20×, sequence length 2.7–3.2 Mb, and contigs ≤300 (Table 1) (11). Reads were assembled using Shovill v1.1.0 (13) and screened for virulence genes using ABRicate v1.0.1 against the Virulence Factor Database (accessed on 5 March 2025) (14), with ≥80% identity and coverage thresholds. Results were summarized with ABRicate Summary v1.0.1 (15) and visualized as heat maps in the Interactive Tree of Life (16).

**TABLE 1:** Metadata and quality metrics for sequenced Listeria monocytogenes isolates

Isolate strain	SRA accession no.	Location^*a*^	Collection date	Multi-Locus sequence type^*b*^	GC content	Contigs	Length	Coverage	N50	Q-Score R1	Q-Score R2
0102.01	SRR30495538	Downtown	06/04/2024	1648	38%	18	2,927,338	122.5	546,402	34.7	34.6
0101.02	SRR30970138	Downtown	06/13/2024	999	38%	19	2,889,285	48.9	327,225	35.6	34.6
0101.03	SRR30495537	Downtown	06/13/2024	5	38%	28	3,055,372	64.4	476,406	34.7	34.7
0102.04	SRR30970137	Downtown	06/23/2024	5	38%	21	3,048,446	51.7	476,406	35.7	34.7
0103.05	SRR30970135	Downtown	06/23/2024	5	38%	23	3,049,170	45.7	476,636	35.5	34.9
0104.06	SRR30495536	Downtown	06/23/2024	5	38%	32	3,050,344	101.8	476,211	35.1	34.8
0302.07	SRR30495535	UAJA	06/23/2024	1110	38%	18	2,891,474	80	579,317	35	34.4
0302.08	SRR30970134	UAJA	06/23/2024	29	38%	12	2,908,514	55.6	584,212	35.7	34.7
0101.09	SRR30970133	Downtown	07/07/2024	16	38%	17	2,881,595	78.1	581,924	35.6	35.2
0102.10	SRR30970132	Downtown	07/07/2024	5	38%	25	3,055,678	47.5	476,406	35.5	35.2
0301.11	SRR30970131	UAJA	07/07/2024	1996	38%	10	2,921,989	58.4	561,484	35.3	32.7
0102.12	SRR30495534	Downtown	07/11/2024	5	38%	36	3,051,919	65	475,859	35.1	34.9
0301.13	SRR30495533	UAJA	07/11/2024	5	38%	39	3,004,460	61.3	347,844	34.8	34.6
0302.14	SRR30970130	UAJA	07/11/2024	369	38%	14	2,939,247	77.6	511,589	35.6	34.9
0201.15	SRR32311113	Campus	07/14/2024	–	38%	18	2,956,421	55.1	591,849	35.4	32.8
0202.16	SRR30495532	Campus	07/14/2024	–	38%	17	2,886,430	53.4	543,078	34.4	34.8
0301.17	SRR30970129	UAJA	07/14/2024	321	38%	18	2,949,704	88.9	545,164	36	34.6
0302.18	SRR30970128	UAJA	07/14/2024	5	38%	13	3,022,781	85.8	538,355	35.9	34.6
0302.19	SRR30970136	UAJA	07/28/2024	29	38%	15	2,868,158	51.1	543,935	35.4	34.8

^*a*^ Location refers to the sampling site: UAJA (University Area Joint Authority), Campus (Pennsylvania State University’s Water Reclamation Facility), and Downtown (wastewater from downtown State College collected from manhole BD1100 before reaching UAJA facility).

^*b*^ Isolates without a denoted sequence type found through MicroRunQC (v1.2) denoted by "–".

Bacterial Isolate Genome Sequence Database v3 on Institut Pasteur (17) was used to identify clonal complex, lineage, and serovar; all were visualized in iTOL v6 (16). Default parameters were used for all tools unless otherwise specified.

**Fig 1:**
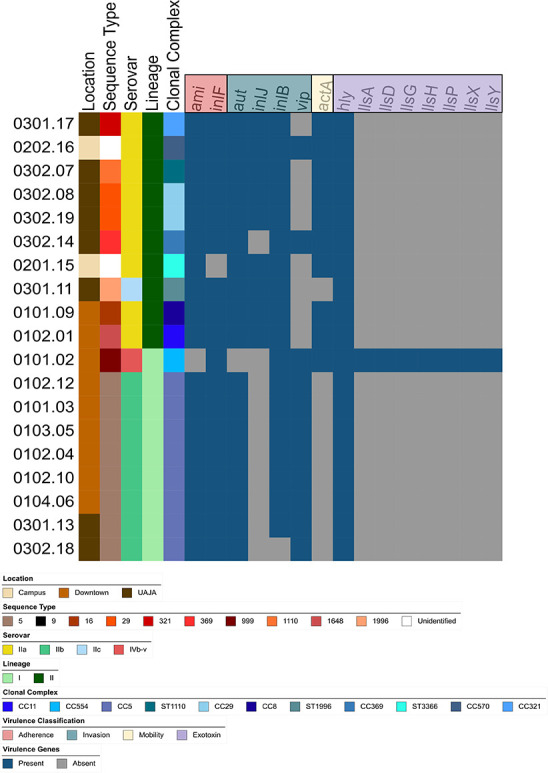
Phylogenetic and virulence gene analysis of *L. monocytogenes* isolates from municipal wastewater. *L. monocytogenes* strains were isolated from three locations: Campus (Pennsylvania State University’s Water Reclamation Facility), Downtown (wastewater from downtown State College, collected at manhole BD1100 before reaching UAJA facility), and UAJA. The color-coded clade legend indicates the sampling location, sequence type, serovar, lineage, clonal complex, and virulence genes identified.

Genomic analyses revealed that one strain (0101.02) belonged to a recently described sequence type (ST999) (Fig. 1) (18). This strain was identified as lineage I, which includes *L. monocytogenes* isolates commonly associated with human illness, and serovar IVb-V (19–21). Strain 0101.02, and two others (0302.08 and 0301.17), differed by ≤9 SNPs from clinical cases available in the NCBI Pathogen Detection Isolates Browser (22). These results suggest genetic similarity to clinical isolates and support the potential of using WBS as a resource for monitoring community levels of foodborne pathogens, as previously demonstrated for *Salmonella enterica* (3, 4, 23).

## Data Availability

The standardized strain descriptions and accession numbers are presented in Table 1; genomic data are publicly available in DDBJ/ENA/GenBank under BioProject no. PRJN357724 and in the Sequence Read Archive under accession no. SRP382070. The versions described are the first versions.
